# Surgical Outcome Prediction Using a Four-Dimensional Planning Simulation System With Finite Element Analysis Incorporating Pre-bent Rods in Adolescent Idiopathic Scoliosis: Simulation for Spatiotemporal Anatomical Correction Technique

**DOI:** 10.3389/fbioe.2021.746902

**Published:** 2021-10-12

**Authors:** Hiroyuki Tachi, Koji Kato, Yuichiro Abe, Terufumi Kokabu, Katsuhisa Yamada, Norimasa Iwasaki, Hideki Sudo

**Affiliations:** ^1^ Department of Orthopaedic Surgery, Faculty of Medicine and Graduate School of Medicine, Hokkaido University, Sapporo, Japan; ^2^ Department of Orthopaedic Surgery, Eniwa Hospital, Eniwa, Japan; ^3^ Department of Systems Information Science, Future University Hakodate, Hakodate, Japan; ^4^ Department of Advanced Medicine for Spine and Spinal Cord Disorders, Faculty of Medicine and Graduate School of Medicine, Hokkaido University, Sapporo, Japan

**Keywords:** surgical outcome, simulation system, finite element analysis, pre-bent rods, adolescent idiopathic scoliosis

## Abstract

An optimal surgical strategy for adolescent idiopathic scoliosis (AIS) is to provide maximal deformity correction while preserving spinal mobile segments as much as possible and obtaining a balanced posture. From a spatiotemporal deformity correction standpoint, we recently showed that anatomical four-dimensional (4D) spinal correction could be accomplished by curving the rod. In the surgical procedure, two rods are bent identically to confirm spinal anatomical alignment without referring to the intraoperative alignment of the deformity. Therefore, anatomically designed rods have been developed as notch-free, pre-bent rods for easier anatomical reconstruction. In addition to providing the best spinal instrumentation configurations as pre-bent rods, prediction of surgical outcome along with its biomechanical impact can be obtained by simulation of the surgical procedures with computer modeling. However, an objective model that can simulate the surgical outcome in patients with AIS has not been completely elucidated. The present study aimed to compare simulated deformity corrections based on our newly developed spatiotemporal morphological 4D planning simulation system incorporating pre-bent rods and actual deformity corrections in patients with AIS. A consecutive series of 47 patients who underwent anatomical posterior correction for AIS curves were prospectively evaluated. After multilevel facetectomy, except for the lowest instrumented segment, 11 types of pre-bent rods were used. Patient demographic data, radiographic measurements, and sagittal rod angles were analyzed within 1 week of surgery. Our simulation system incorporating pre-bent rods showed a significant correlation with the actual postoperative spinal alignment. The present study demonstrated the feasibility of our simulation system and the ability to simulate the surgical procedure using the pre-bent rods. The simulation system can be used to minimize the differences between the optimal and possible outcomes related to the instrumentation levels and rod shapes. Preoperative assumption of rod shape and length can contribute to a reduction in operative time which decreases blood loss and risk of infection. The results of the finite element analysis in the simulation system measured for each individual patient would also provide a more realistic representation of the surgical procedures.

## Introduction

Adolescent idiopathic scoliosis (AIS) is the most commonly encountered pediatric musculoskeletal disease presenting a three-dimensional (3D) deformity of the spine. Standard measurement in scoliosis is the Cobb angle, which is the coronal plane angle measured between the vertebrae at the upper and lower bounds of the curve on a standing radiograph. Patients with severe (Cobb angle >40°–50°) or progressive curves may require surgery to correct the deformity.

An optimal surgical strategy is to provide maximal deformity correction while preserving spinal mobile segments to the best extent and obtaining a balanced posture. For instance, inadequate selection for instrumentation length may lead to a postoperative postural imbalance. In addition, although surgical technique as well as spinal instrumentation has been developed in which the 3D correction is achieved, there is still a possibility of implant-related complications such as pedicle screw loosening, screw or spinal rod breakage, and pedicle fracture. Load levels of the screws and rods are important concerns in surgical outcomes. Furthermore, although a rod shape considerably affects postoperative spinal alignment (Salmingo et al., 2014; Kokabu et al., 2016; Sudo et al., 2016; Le Navéaux, et al., 2017), the rod-bending maneuver relies excessively on surgeons’ experience. If the rod curvature does not match the patient’s deformity and does not allow for deformity correction, such situations will lead to an inadequate correction or implant-related complications due to the overstress on the implant and spine (Sudo et al., 2018). These issues require some innovative systems to assist surgery or predict the most probable outcome of surgery (Aubin et al., 2008; Sudo et al., 2021).

**FIGURE 1:**
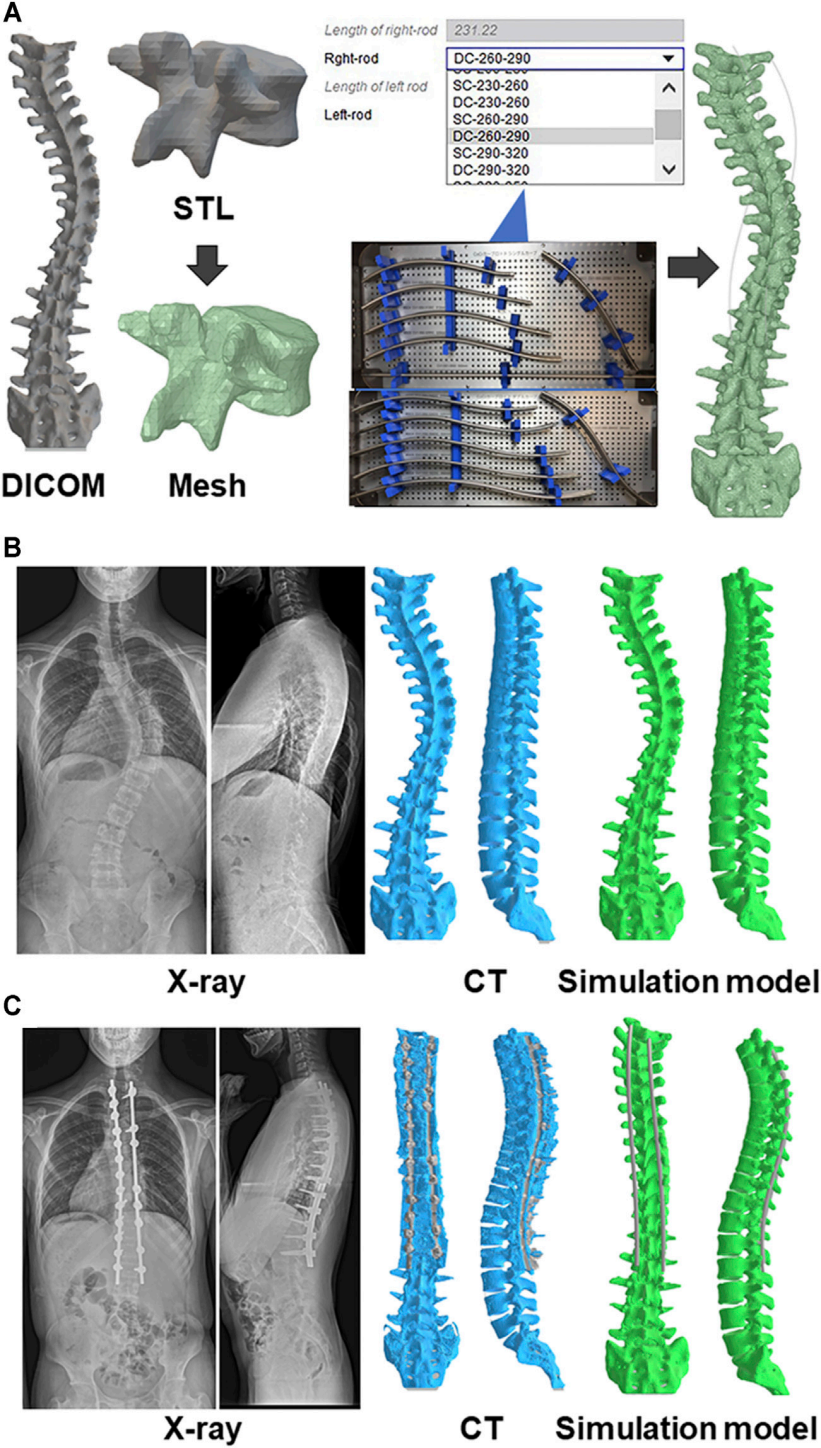
Biomechanical model of the spine **(A)** A custom spinal finite element model was constructed based on the preoperative computed tomography (CT) data. The collected raw data in the Digital Imaging and Communications in Medicine (DICOM) format were imported in a standard triangle language (STL). Subsequently, the STL data were converted to a solid structure, which were built of 10-node tetrahedral element meshes. Eleven types of beam element rods were selected based on the deformity types and its lengths, and positioned for the screws. Representative pre**(B)**- and post**(C)**operative images of radiograph, CT, and simulation model.

The typical thoracic AIS presents itself with thoracic hypokyphosis. Therefore, the surgical goal should be a correction of the thoracic kyphosis (TK) and achieves an anatomically correct thoracic curve. Post-surgery hypokyphosis can occur after using pedicle screw instrumentation. Several posterior surgical techniques have been developed to maintain and/or improve the TK (Clement et al., 2008; Sudo et al., 2014). However, next-generation surgical techniques are required in order to achieve true anatomical correction. In a healthy human population, the apex of the TK is typically located at T6–T8, when viewing standing sagittal films (Hasegawa et al., 2017). However, for some AIS, the postoperative apex of the TK is almost identical with the apex of the preoperative thoracic scoliosis (Sudo et al., 2018), which is not anatomically correct. This insufficient correction resulting in a postoperative non-anatomical TK is thought to be, because the spinal rods are being bent to match the curvature of scoliosis. From the standpoint of spatiotemporal deformity correction, we recently showed that anatomical four-dimensional (4D) spinal correction could be accomplished by curving the rod (Figure 1) (Sudo et al., 2018; Sudo et al., 2021). In the surgical procedure, two rods are bent in a nearly identical fashion to confirm spinal anatomical alignment without reference to the intraoperative alignment of the deformity (Sudo et al., 2018; Sudo et al., 2021). Consequently, pre-bent rod geometries were obtained from intraoperative tracings of the rod shapes, and optimized rod shapes were derived using iterative closest point method followed by hierarchical cluster analysis (Kokabu et al., 2018). Currently, 11 types of pre-bent cobalt-chrome (CoCr) alloy rods are available based on the deformity types and its lengths in Japan that can guide anatomical spinal correction regardless of the surgeons’ experience (Figure 1, Sudo et al., 2021).

In addition to providing the best spinal instrumentation configurations as pre-bent rods, prediction of surgical outcome along with its biomechanical impact can be obtained by simulation of the surgical procedures with computer modeling (Aubin et al., 2008). However, an objective model that can simulate the 3D outcome of the AIS surgery by considering the preoperative spinal alignment and the surgical intervention has not been completely elucidated (Pasha and Flynn, 2018). In addition, most planning tools in AIS surgery only simulate morphology-based changes of the spinal alignment, lacking the biomechanical analysis (Ferrero et al., 2008; Pasha and Flynn, 2018; Shao et al., 2018). A planning simulator based on spatiotemporal morphological postoperative 4D changes with a patient-specific finite element analysis (FEA) can allow surgeons to predict postoperative outcomes and effectively assist in performing AIS surgery (Galbusera et al., 2015; Wang et al., 2016; Le Navéaux et al., 2016; Cobetto et al., 2020; La Barbera et al., 2021; Galbusera et al., 2021).

The hypothesis of the present study was that our newly developed 4D planning simulation system incorporating pre-bent rods would significantly correlate with the actual postoperative spinal alignment after anatomical 4D spinal correction surgery. The current study aimed to compare simulated and actual deformity corrections in patients with AIS.

## Materials and Methods

### Patient Selection

After institutional review board approval, data from a consecutive series of 47 patients who underwent 4D anatomical correction surgery for AIS curves between 2019 and 2021 were prospectively evaluated; all patients had a Cobb angle of ≤90°. We did not define the lower limit of the Cobb angle. However, patients with severe (Cobb angle >40°–50°) and/or progressive curves were included. The Ethics Committee of Hokkaido University Hospital approved this research including any relevant details. All methods were performed in accordance with the relevant guidelines and regulations. Written consents were obtained from all the subjects, and when applicable from their guardians. The exclusion criteria were neuromuscular, congenital, and other syndromic scoliosis.

Standing posteroanterior radiographs were recorded preoperatively and within 1 week after surgery. Regarding Cobb measurements, the end vertebrae levels were determined on preoperative radiographs and measured on subsequent radiographs to maintain consistency for statistical comparisons (Cidambi et al., 2012; Sudo et al., 2013). The angle of rotation of the main thoracic (MT) and/or thoracolumbar/lumbar (TL/L) apical vertebra was determined on computed tomography (CT) images (Cidambi et al., 2012; Silvestre et al., 2013; Sudo et al., 2014). Internal studies of the present interrater and intrarater reliability have demonstrated high kappa statistics for all continuous measures (0.90–0.98).

### Surgical Procedures

While the end vertebrae were to be considered part of the instrumentation levels, the selection of the upper or lower instrumented vertebrae was dependent on several preoperative anatomical conditions. Shoulder balance and anatomical TK determine the vertebra that was selected for the upper instrumented vertebra (UIV); T2 was selected if the radiographic shoulder height (RSH) was positive, T3 if RSH was between −5 and 0 mm, and T4 if RSH was < −5 mm (Sudo et al., 2018). However, in case with TK < 20° and T5 or T6 upper-end vertebra, the UIV selected was T4 to create anatomical TK (Sudo et al., 2018). The lowest instrumented vertebra (LIV) depends on the lumbar modifiers. For a lumbar modifier A or B, the last vertebra touching the center sacral vertebral line was the LIV (Matsumoto et al., 2013). In the case of lumbar modifier C, LIV was determined at L3 (Sudo et al., 2021).

Side-loading polyaxial pedicle screws (CVS spinal system; Robert Reid, Tokyo, Japan) were inserted. Our previous studies have shown that multilevel facetectomy and screw density on the concave side rather than the convex side significantly impact scoliosis correction and TK restoration (Sudo et al., 2016). This means it is important to place as many screws as possible on the concave side. On the convex side, screws should be placed at 1) the UIV, 2) the upper-end vertebra, 3) the lower-end vertebra, 4) the LIV, and 5) at the apex of scoliosis and its periapical lesions. All-level facetectomy was performed in all patients except for the lowest instrumented segment to avoid pseudoarthrosis at this site (Sudo et al., 2018). For pre-bent rods, CoCr alloy rods (φ 5.5 mm) were bent identically to duplicate the postoperative anatomical TK (Sudo et al., 2018; Sudo et al., 2021). The apex was anticipated to be at T6–T8 for the postoperative TK (Sudo et al., 2018). The rod configurations were split into two types of shapes: single curve and double curves. In the case that LIV was L1 or above, the single-curve rods were applied (Sudo et al., 2018; Sudo et al., 2021), and the TL/L region remained straight. When the LIV was L2 or L3, the double-curve rods were applied (Sudo et al., 2018; Sudo et al., 2021). Each shape was provided by increments of 3 cm. After connecting to the screw heads, the rods were simultaneously rotated. During the rod derotation maneuver, the present technique helped prevent the hypokyphotic deformation of the rod compared with the simple single-rod derotation maneuver or direct vertebral rotation technique (Sudo et al., 2014). The simultaneous rod rotation maneuver does not intend to manipulate vertebral rotation at each level separately and works to correct the rotational deformity not at each segment separately but in the entire instrumentation area simultaneously (Sudo et al., 2014). After 90° rod rotation, several screw heads were tightened to lock the rods. The presence of a mark on the rod helped confirm 90° rotation. Distraction force was first applied on each screw head on the concave side of the thoracic curve, so that not only scoliosis but also TK could be corrected more effectively by lengthening the posterior column. Subsequently, compression force was applied segmentally on the convex curve. *In situ* rod-bending procedure was not performed (Sudo et al., 2014; Sudo et al., 2016; Sudo et al., 2018).

### Biomechanical Model of the Spine

For each patient, a custom spinal finite element model (FEM) was constructed based on the preoperative CT Digital Imaging and Communications in Medicine (DICOM) data (Figure 1). The software ANSYS 19.2 (ANSYS JAPAN, Tokyo, Japan) was used to model the spine and perform surgical simulations. The collected raw data in the DICOM format were imported into Mimics research 19.0 (Materialize, Leuven, Belgium) to generate 3D vertebral models in a standard triangle language (STL). Subsequently, the STL data generated were imported into ANSYS 19.2 in the form of solid 3D structure (Peng et al., 2020; Zhou et al., 2020), which was built of 10-node tetrahedral element meshes (Ulrich et al., 1998). Screws were positioned and oriented in the desired locations. A new triangulated surface of the instrumented vertebra was generated by Boolean subtraction between the original vertebral surface and the surface of the screws to represent the insertion of screws into the vertebrae. Tetrahedral finite element meshes of the vertebrae and screws were then automatically generated (Galbusera et al., 2015). Eleven types of beam element rods were selected based on the deformity types and its lengths, and positioned for the screws.

Vertebrae were considered as rigid elements in the model to avoid penetration of bone structures. Spinal rods were modeled with a cross-sectional diameter of 5.5 mm, Young’s modulus of 420 GPa, and Poisson’s ratio of 0.3 according to CoCr alloy properties (Yamada et al., 2020). The screws were modeled as cylinders with a length of 30 mm, cross-sectional diameter of 5.5 mm, Young’s modulus of 5,000 MPa, and Poisson’s ratio of 0.3 (Zhou et al., 2020). Rods and screws were modeled with materials of isotropic elastic linear material properties. The number of elements in the implants was 790 in the rod and 88 in each screw (Shin et al., 2018).

Connections between the geometries were defined to simulate spatiotemporal morphological postoperative 4D changes. For stability of simulation, intervertebral discs were completely removed, and each vertebra was connected with joint element having intervertebral stiffness (Galbusera et al., 2015). The stiffness matrix components used in this study (Table 1) were using the ANSYS software. We set the stiffness matrix components based on the literature (Argoubi and Shirazi-Adi, 1996). The intervertebral stiffness was calculated from reaction forces and moments according to the relative amount of translational and rotational displacements between two vertebrae (Senteler et al., 2016). The values were same through T1/2 to L4/5. However, the value of stiffness matrix component was reduced to be representative of facetectomy (Oda et al., 2002) where facetectomy was performed. The connection between vertebra and screw was set as a joint where boundary condition was defined with all translational and rotational degrees constrained. A spring contact model was defined between screw head and rod to simulate the rod being captured in the screws. Finally, a spring contact model was defined between the concave and convex rods to simulate the rotation of rods simultaneously. In this study, distractioncompression force was not applied on each screw head.

**TABLE 1:** Stiffness matrix components used in this study.

	Force [N/mm] (with facetectomy)			Moment [N・mm/deg] (with facetectomy)		
	Antero-posterior	Medio-lateral	Cranial-caudal	Antero-posterior	Medio-lateral	Cranial-caudal
T1/2-L4/5	1,392 (696)	294 (188)	341 (188)	448 (224)	644 (406)	738 (406)
L5/S	700	190	190	222	410	410

### Surgical Simulations

During the whole simulation process, boundary conditions were imposed on the spinal FEM to mimic conditions observed in a surgical setting and ensure simulation convergence. Boundary conditions of the spinal FEM were defined with the sacrum fixed and T1 free to rotate and to translate in the caudocranial direction, allowing possible lengthening of the spine during the simulation of the correction process. For multilevel facetectomy simulation, contacts between posterior facets were neglected at instrumented levels except for the lowest instrumented segment to mimic their surgical removal. Regarding rod rotation maneuver, connecting concave and convex rods to screw head was simulated by setting the spring length between the rod and the screw to zero. Furthermore, the power delivered to the concave rod was gradually increased towards concave (66 ± 106 N; range, 0–300 N) and dorsal sides of scoliosis (922 ± 169 N; range, 400–1,000 N) to perform a 90° rotation of the rod. Then, final locking of the screws was simulated. For each screw, null relative translations and rotations between the appropriate rod node and the screw head were imposed. Therefore, the rods could not slide or rotate anymore into the screw heads. Moreover, all external constraints (displacement or forces) were released at this step, and the new equilibrium state was computed. The model was left free to reach equilibrium at the end of the simulation. In this surgical simulation, distractioncompression force was not applied on each screw head. Hence, intraoperative surgical steps described in the simulation included precisely the same as actual surgery except for distractioncompression procedure and screw length and diameter. However, the stiffness matrix does not correctly describe the real condition because this model did not consider preoperative curve flexibility.

### Simulation Data Analysis

The spinal profile, quantified in terms of coronal MT Cobb angle, TK and TL/L lordosis, and apical vertebral rotation angle, was monitored over the course of the surgery simulation process. Von Mises stress, which is an equivalent stress, was shown as the reaction forces on the concave and convex rods at the end of the correction (Shin et al., 2018; Peng et al., 2020). Axial forces at the bonescrew interface were analyzed for all the pedicle screws with respect to their simulated final positions and the postoperative ones. The direction of these forces was along the direction of the screw’s shaft. They were supposed to be responsible for the pull-out phenomenon; therefore, the expression “pull-out forces” was used to describe them. This first phase study did not analyze other forces and moments components such as medio-lateral forces and screw bending moments (responsible for pedicle wall breach and screw breakage, respectively) because the pull-out phenomenon is likely observed compared to the other phenomena in actual AIS surgery (Abul-Kasim and Ohlin, 2014; Oda et al., 2021).

### Analysis of Rod Configuration

**FIGURE 2:**
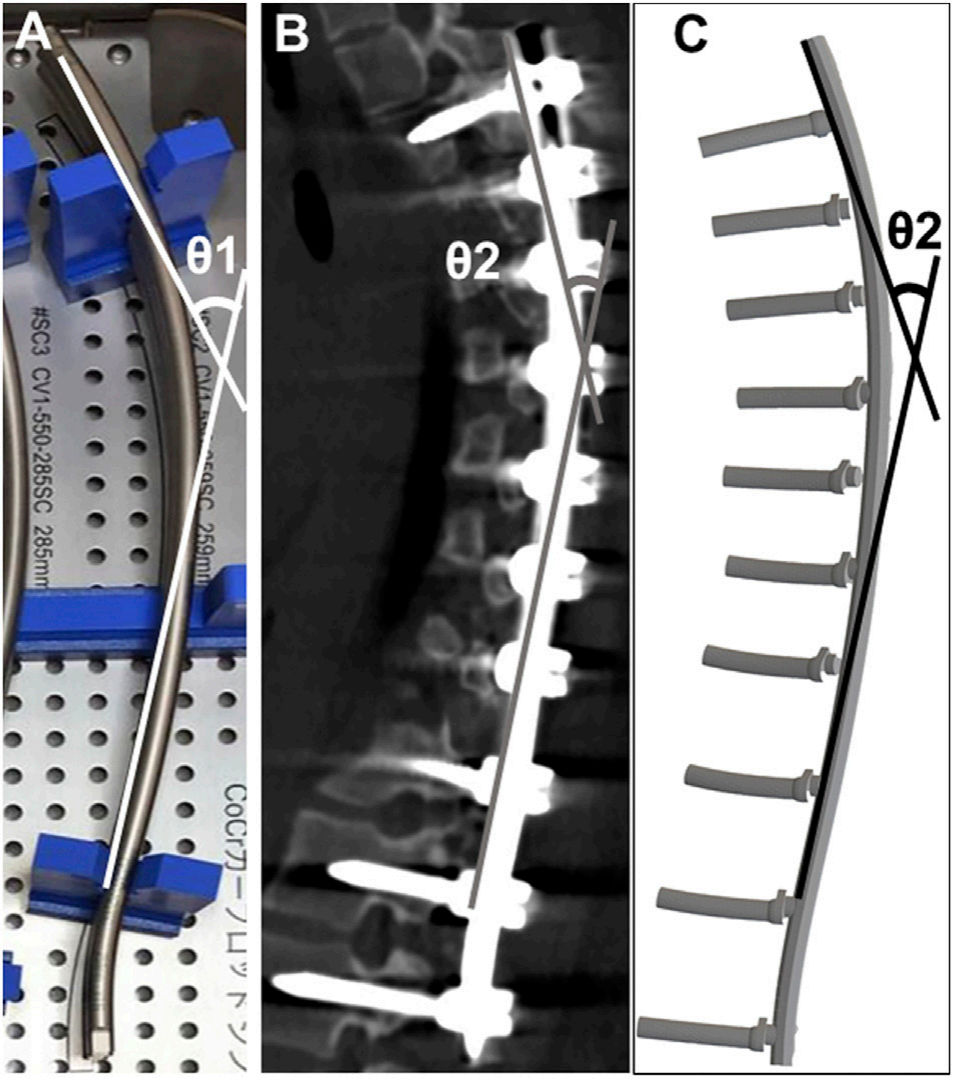
Rod angle before and after implantation **(A)** Prior to implantation, the angle between the proximal and distal tangential line was measured (θ1). Postoperative implant rod geometry (θ2) was obtained after the surgery using computed tomography **(B)** and the simulation model **(C)**.

The angle between the cranial and caudal tangential lines was obtained before implantation (θ1) (Figure 2). Similarly, the postoperative rod angle was obtained (θ2) using reconstructed sagittal CT images and simulation models (Cidambi et al., 2012; Kokabu et al., 2016; Sudo et al., 2021). The angle of rod deformation was defined as the difference between θ1 and θ2 (θ1–θ2) (Kokabu et al., 2016; Sudo et al., 2021).

### Statistical Analysis

All data were presented as means ± standard deviation and range. The entire cohort was first analyzed and then further assessed based on selective thoracic fusion to L1 in ten Lenke 1 A patients to confirm the feasibility of our simulation system and the ability to simulate the uninstrumented lumbar segments. Repeated-measures analysis of variance was used to compare differences among the standing radiographs, CT images, and simulation data. Data were checked for normality and equality of variances, and Bonferroni post-hoc analysis was used to set the significance level at 0.05. Comparisons of radiographic quantitative variables and rod angles were performed using MannWhitney *U* test or paired *t*-test. Spearman’s correlation coefficient analysis was used to assess relationships between CT images and simulation models. Data analyses were performed using JMP statistical software for Windows (version 14; SAS, Inc, Cary, NC, United States). *p* < 0.05 was considered statistically significant.

## Results

### Patient Demographic Data

Demographic data are summarized in Tables 2, 3. The cephalad-instrumented vertebrae ranged from T2 to T6, and the caudal-instrumented vertebrae ranged from T12 to L3. Preoperative standing radiographic MT and TL/L curves averaged 52° and 37°, respectively, and TK angle was 17°, whereas the lumbar lordosis angle was of 47°. Postoperatively, MT and TL/L curves averaged 11° and 9°, respectively, and TK angle was 30°, whereas the lumbar lordosis angle was 48°. The average MT and TL/L curve correction rate was 81% (range, 64–98%) and 75% (range, 36–98%), respectively. The average preoperative MT and TL/L vertebral rotation angles measured on CT images were 18° each, which decreased after surgery to an average of 11° each.

**TABLE 2:** Patient demographic data.

	Mean ± standard deviation (range)
Number of patients	47
Age at surgery (yr.)	14.7 ± 2.5 (10–19)
Gender (no. and % of woman)	42 (89%)
Risser sign (grade)	3.9 ± 1.3 (0–5)
Lenke type (no.)	
1	31
2	3
3	1
4	1
5	5
6	6
Lumbar modifier (no.)	
A	23
B	3
C	21
Number of instrumented vertebrae (segments)	11.2 ± 1.5 (8–14)

**TABLE 3:** Radiographic and CT parameters.

	Standing radiographs (range)	CT (range)	Simulation model (range)	Repeated-measures analysis of variance	Bonferroni *p*		
				*p*	Standing radiographs to CT	Standing radiographs to simulation model	CT to simulation model
Preoperative coronal plane data							
Main thoracic curve (°)	52 ± 11 (28–82)	45 ± 11 (20–83)	45 ± 11 (20–83)	<0.001	0.011	0.011	1.000
Thoracolumbar/lumbar curve (°)	37 ± 13 (16–72)	32 ± 13 (8–65)	32 ± 13 (8–65)	0.008	0.022	0.022	1.000
Preoperative sagittal plane data							
Thoracic kyphosis (°)	17 ± 9 (2–42)	13 ± 7 (3–32)	13 ± 7 (3–32)	0.037	0.042	0.042	1.000
Lumbar lordosis (°)	47 ± 10 (18–69)	42 ± 10 (24–72)	42 ± 10 (24–72)	0.012	0.024	0.024	1.000
Preoperative vertebral rotation angle							
Main thoracic apical vertebra (°)	NA	18 ± 8 (3–35)	18 ± 8 (3–35)	1.000			
Thoracolumbar/lumbar apical vertebra (°)	NA	18 ± 9 (4–41)	18 ± 9 (4–41)	1.000			
Postoperative coronal plane data							
Main thoracic curve (°)	11 ± 7 (1–28)	13 ± 6 (3–30)	14 ± 6 (2–30)	0.916			
Thoracolumbar/lumbar curve (°)	9 ± 6 (1–27)	12 ± 8 (1–30)	11 ± 6 (2–26)	0.782			
Postoperative sagittal plane data							
Thoracic kyphosis (°)	30 ± 4 (19–38)	28 ± 4 (20–39)	28 ± 4 (20–36)	0.632			
Lumbar lordosis (°)	48 ± 9 (36–69)	48 ± 8 (35–68)	47 ± 7 (33–65)	0.811			
Postoperative vertebral rotation angle							
Main thoracic apical vertebra (°)	NA	11 ± 6 (1–21)	11 ± 6 (0–23)	0.719			
Thoracolumbar/lumbar apical vertebra (°)	NA	11 ± 7 (1–30)	10 ± 6 (1–26)	1.000			

All data expressed as means ± SD and range.

### Comparison Among Standing Radiographs, CT Images, and Simulation Models

Preoperatively, there were significant differences between standing radiographs and CT images or simulation models in both coronal and sagittal plane data (*p* < 0.05). However, there were no significant differences among postoperative standing radiographs, CT images, and simulation models in both coronal and sagittal plane data (*p* > 0.05). Regarding postoperative vertebral rotation angle, there was no significant difference between CT images and simulation model (*p* > 0.05).

### Correlation Analysis and Accuracy Evaluation

**FIGURE 3:**
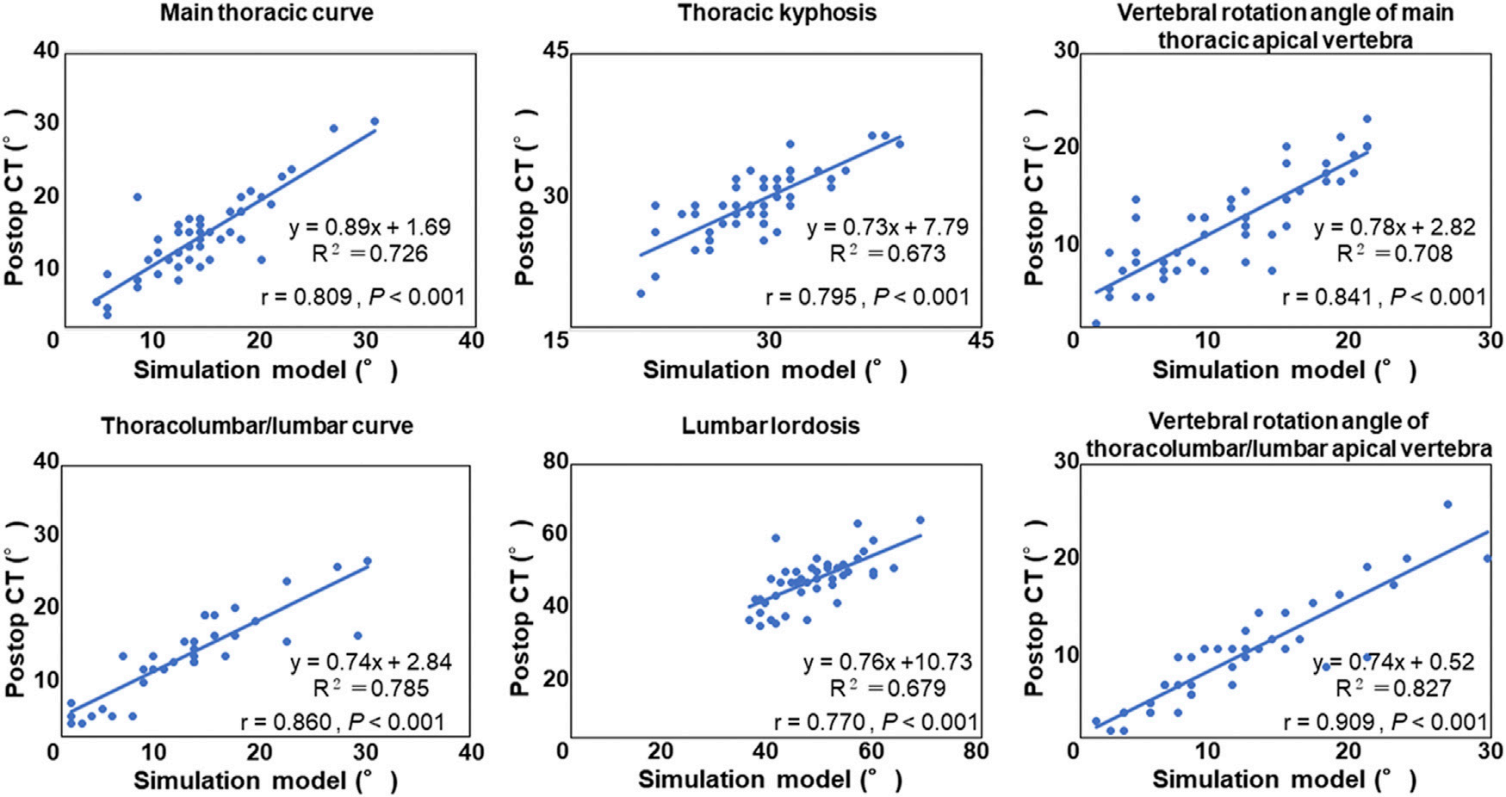
Correlation analysis between the postoperative computed tomography (CT) measurement and the simulation model.

Spearman’s correlation coefficient analysis showed that simulated coronal and sagittal plane data as well as vertebral rotation angle were significantly correlated with those of data on postoperative CT images (*p* < 0.001, Figure 3). Mean absolute error and root mean squared error between CT images and simulation model are summarized in Table 4. Simulated coronal and sagittal plane data as well as vertebral rotation angle were predicted within 5° compared to actual postoperative measurements.

**TABLE 4:** MAE and RMSE between CT images and simulation model.

	MAE	RMSE
Coronal plane data		
Main thoracic curve (°)	3.1	4.1
Thoracolumbar/lumbar curve (°)	2.3	3.5
Sagittal plane data		
Thoracic kyphosis (°)	2.0	2.4
Lumbar lordosis (°)	3.7	4.7
Vertebral rotation angle		
Main thoracic apical vertebra (°)	2.5	3.3
Thoracolumbar/lumbar apical vertebra (°)	2.7	3.6

MAE, mean absolute error; RMSE, root mean squared error.

### Subgroup Analysis

There were no significant differences between postoperative CT images and simulation models in all coronal, sagittal, and axial plane data in Lenke 1 A patients (Table 5, *p* > 0.05). Regarding mean absolute error and root mean squared error between CT images and simulation model, the simulated coronal and sagittal plane data as well as vertebral rotation angle were predicted within 5° compared to actual postoperative measurements (Table 6).

**TABLE 5:** Postoperative CT and simulation model parameters in patients with Lenke type 1 A.

	CT (range)	Simulation model (range)	*p*
Coronal plane data			
Main thoracic curve (°)	14 ± 5 (3–22)	14 ± 6 (4–23)	0.909
Thoracolumbar/lumbar curve (°)	9 ± 5 (1–16)	8 ± 4 (3–12)	0.629
Sagittal plane data			
Thoracic kyphosis (°)	28 ± 3 (24–31)	28 ± 3 (23–32)	0.848
Lumbar lordosis (°)	48 ± 9 (37–68)	49 ± 6 (41–64)	1.000
Vertebral rotation angle			
Main thoracic apical vertebra (°)	13 ± 5 (6–20)	12 ± 4 (5–19)	0.424
Thoracolumbar/lumbar apical vertebra (°)	6 ± 3 (1–12)	5 ± 3 (1–10)	0.445

All data expressed as means ± SD and range.

**TABLE 6:** MAE and RMSE between CT images and simulation model.

	MAE	RMSE
Coronal plane data		
Main thoracic curve (°)	2.1	3.4
Thoracolumbar/lumbar curve (°)	2.0	2.4
Sagittal plane data		
Thoracic kyphosis (°)	1.4	1.7
Lumbar lordosis (°)	3.5	4.3
Vertebral rotation angle		
Main thoracic apical vertebra (°)	1.6	2.0
Thoracolumbar/lumbar apical vertebra (°)	1.4	1.7

In patients with Lenke type 1 A.

MAE, mean absolute error; RMSE, root mean squared error.

### Analysis of Rod Stress and Screw Forces

**FIGURE 4:**
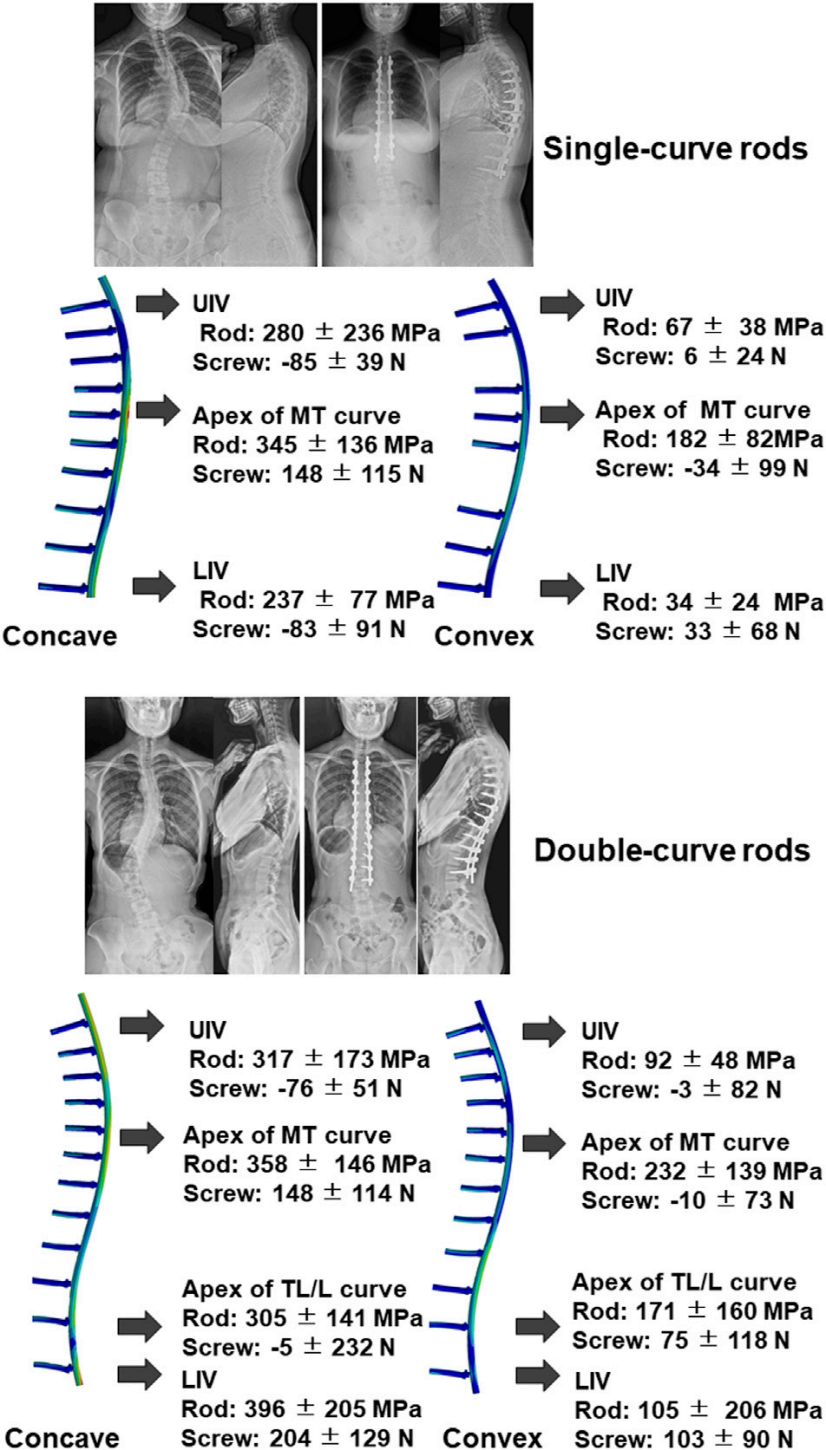
Representative images of radiograph and simulation model using single-curve rod **(upper)** and double-curve rods. Results of rod stress and screw forces analysis of all 47 cases were also presented. Positive value in the screw force means pull-out force. UIV; upper instrumented vertebra, MT; main thoracic, LIV; lowest instrumented vertebra, TL/L; thoracolumbar/lumbar.

We estimated rod stress and screw force in simulation models. The models showed that peak stress was located near the apex of the curve in both single and double curve type rods (Figure 4). In addition, another peak was located near the extremities of the instrumented segments. Table 7 shows estimated rod stress and screw force in one simulation model. The rod stress was significantly higher at the concave side compared to the convex side (*p* < 0.05). Regarding pull-out forces on screws, peak forces were located near the apex of the MT curve at the concave side in both single and double curves. In addition, another peak was located near the LIV at both concave and convex sides in double curve. The pull-out forces on screws were significantly higher at the concave side compared to the convex side (*p* < 0.05).

**TABLE 7:** Estimated rod stress (MPa) and screw forces (N) in simulation model.

	Rods			Screws		
	Concave	Convex	*p*	Concave	Convex	*p*
Single-curve rods (N = 15)						
UIV	280 ± 236 (52–931)	67 ± 38 (21–172)	0.002	−85 ± 39 (−179 to −20)	6 ± 24 (−33–57)	<0.001
Apex of MT curve	345 ± 136 (127–527)	182 ± 82 (24–312)	0.001	148 ± 115 (48–432)	−34 ± 99 (−220 to 220)	<0.001
LIV	233 ± 77 (113–358)	34 ± 24 (11–109)	<0.001	−83 ± 91 (−239 to 95)	33 ± 68 (−52–214)	0.004
Double-curve rods (N = 32)						
UIV	317 ± 173 (105–856)	92 ± 48 (20–220)	<0.001	−76 ± 51 (−188 to 35)	−3 ± 82 (−157–224)	<0.001
Apex of MT curve	358 ± 146 (69–840)	232 ± 139 (43–580)	<0.001	148 ± 114 (−134–416)	−10 ± 73 (−242 to 135)	<0.001
Apex of TL/L curve	305 ± 141 (126–586)	171 ± 160 (21–773)	<0.001	−5 ± 232 (−411 to 395)	75 ± 118 (−153–307)	0.109
LIV	396 ± 205 (50–1,002)	105 ± 206 (43–580)	<0.001	204 ± 129 (7–497)	103 ± 90 (−60–307)	0.001

All data expressed as means ± SD and range. Positive value in the screw force means pull-out force. UIV, upper instrumented vertebra; MT, main thoracic; LIV, lowest instrumented vertebra, TL/L, thoracolumbar/lumbar.

### Implant-Rod Angles of Curvature

The rod deformation angle was significantly higher on the concave side than on the convex side (*p* < 0.001; Table 8). There were no significant differences between postoperative CT images and simulation model in the rod deformation angle at both concave and convex sides (*p* = 0.129 and *p* = 0.237, respectively).

**TABLE 8:** Implant-rod angle of curvature.

	Concave	Convex	*p*
Preoperative rod angle (θ1) (°)			
CT	39.3 ± 5.7 (29.1–46.1)	39.3 ± 5.7 (29.1–46.1)	1.000
Simulation model	39.3 ± 5.7 (29.1–46.1)	39.3 ± 5.7 (29.1–46.1)	1.000
Postoperative rod angle (θ2) (°)			
CT	32.2 ± 4.2 (24.2–40.0)	36.2 ± 4.9 (27.2–43.0)	<0.001
Simulation model	33.2 ± 4.2 (26.8–41.3)	36.8 ± 5.0 (28.0–44.7)	<0.001
Rod deformation angle (Δθ) (°)			
CT	7.1 ± 3.1 (1.3–13.8)	3.2 ± 2.5 (−1.8–8.9)	<0.001
Simulation model	6.5 ± 3.2 (0.2–12.6)	2.5 ± 2.9 (−3.9–7.6)	<0.001

All data expressed as means ± SD and range. Δθ was defined as the difference between θ1 and θ2 (θ1–θ2).

## Discussion

Our personalized finite element spinal biomechanical model and its simulated response to surgical instrumentation allowed to evaluate the effect of the 4D anatomical correction technique on AIS deformity correction and on loads in the instrumentation. Not only geometric aspects of the deformity correction but also biomechanical results were simulated using existing pre- and postoperative information of 47 patients and 11 types of pre-bent rods. Despite the heterogeneity in the cohort of patients with various Lenke types, this study analyzed a cohort of patient-specific surgery models and found that our newly developed 4D planning simulation system incorporating pre-bent rods showed a significant correlation with the actual postoperative spinal alignment after anatomical 4D spinal correction surgery. The simulated measurements were all within 5° agreement with the clinical values, equivalent to the generally accepted clinical error of 5° (Majdouline et al., 2009).

The present study demonstrated the feasibility of our simulation system and the ability to simulate the surgical procedure using the pre-bent rods. The preoperative assumption for rod shape and length will help reduce operative time, thereby decreasing blood loss and risk of infection. However, in this model, the values of intervertebral stiffness matrix components were same through thoracic to lumbar spines and not personalized. We consider this as a main factor that may justify imperfect correlations of the slope and intercept. It may be important to consider the personal stiffness matrix for improving the capability of the model in predicting the perfect values. Other factors affecting the correlations could be that boundary conditions applied during simulation were not the same as during the actual surgery for each patient, and there was an issue that clinical measurements of the parameters of interest were not perfect. In addition, we cannot provide a quantitative justification regarding the prediction accuracy because no steps were used to ensure the credibility of our model in predicting the right values. Although computational models are increasingly used to support surgical planning, varying levels of model verification and validation limit the level of confidence in their predictive potential (Poncelas et al., 2021). Recently, Poncelas et al. performed a credibility assessment of their model to investigate proximal junctional failure in clinical cases with adult spine deformity using ASMEV&V40 standard (Poncelas et al., 2021). We should also assess the credibility of our model for AIS surgery using the recommended strategies in the future.

In the present study, the surgical simulations were conducted using the DICOM CT scans in a supine position and approximated with the surgical procedures performed on patients lying prone. In addition, the postoperative corrected angles were measured clinically using the DICOM CT data in a supine position. Consequently, supine CT scans were obtained after surgery, while the patient was still recovering; therefore, it was not yet load-bearing, and provided a better comparison between the clinical and simulated measurements (Little et al., 2013). The difference between the present simulation model and reality was small in the instrumented segments. Conversely, there is a possibility that the results for the uninstrumented regions are not accurate because the gravity and postural control were not simulated in the standing posture (Robitaille et al., 2009). Due to the aforementioned reasons, we performed comparisons using standing radiographs. Consequently, while there were significant differences in preoperative measurement values between standing radiographs and simulation model, there were no significant differences in postoperative measurement values between the standing radiographs and simulation model, indicating that the simulation model can predict postoperative spinal alignment in standing position including uninstrumented lumbar segments in the case with selective thoracic instrumentation for Lenke 1 A curves.

There have been computational studies that similarly predicted 3D correction and implant loads (Wang et al., 2016; Le Navéaux et al., 2016; La Barbera et al., 2021; Galbusera et al., 2021). These studies determined how several instrumentation parameters such as screw density and rod contouring angle affected correction and stress in the instrumentation. Because our previous studies have shown that multilevel facetectomy and screw density on the concave side significantly impact the amount of scoliosis correction and also TK restoration, especially in preoperative hypokyphotic (TK <15°) thoracic spine (Kokabu et al., 2016; Sudo et al., 2016), we currently attempt on inserting the screw as much as possible on the concave side. Also, rod curvatures were limited to up to 11 types. We have analyzed the correlation between preoperative rod angle and rod stress in the apex of the MT curve or TL/L curve. In addition, the correlation between postoperative TK in the simulation model and screw density on the concave side or preoperative rod angle in patients with hypothoracic (TK <15°) Lenke one curves was analyzed. The results showed that there were no significant correlations between the simulated correction and rod stress and instrumentation parameters (Supplementary figures S1, S2). Therefore, we could not confirm that clinical observation reported by Sudo et al. (2016) and Kokabu et al. (2016) was confirmed by the present biomechanical models. Because there was no range of numbers for the screw density and rod curvature, as well as sufficient quantity of sample numbers, there may be limitations in statistical analysis.

However, because both pre- and postoperative rod measurements were available in the current study, it was possible to utilize the initial rod shape and simulate its elastic deformation precisely. In this simulation model, the maximum power delivered to the rod was 1,000 N. We previously documented that the notch-free, pre-bent CoCr alloy rod (φ5.5 mm) showed an approximated linear loaddisplacement curve under 1000 N of load (Yamada et al., 2020). Due to these reasons, we opine that almost only elastic deformation occurred. However, elastoplastic phenomena involved in rod contouring may be considered in future studies to better elucidate whether both elastic and plastic deformation may be involved *in vivo* to explain any change in rod shape *in vivo*. Although it has been demonstrated that the amount of curvature incorporated into the rods before their insertion impacts TK restoration (Salmingo et al., 2014; Kokabu et al., 2016; Sudo et al., 2016; Le Navéaux, et al., 2017), there are a few studies that have estimated the loads in the rod during and after deformity collection (Galbusera et al., 2015). To our knowledge, this is the first study to show that the simulation model can predict the deformation of the implanted rod. The simulation model determined significant changes in the rod contours, especially on the concave side which has been clinically reported (Sudo et al., 2021). In addition, based on the changes in rod geometry and FEA, the highest stress was found at the apex of the rod curvature and the extremities of the instrumented levels, which is in agreement with previous results (Belmont et al., 2001; Aubin et al., 2008; Abe et al., 2015). Qualitative understanding of the stress in the rods is useful to estimate the risk of implant failure and loosening intraoperatively and/or postoperatively, which currently depends on the surgeon’s experience (Galbusera et al., 2015).

Further understanding of bonescrew forces in AIS instrumentation is essential as high-stresses at the bonescrew interface can cause screw loosening or breakage. Shear forces on the screws are more relevant to be reported rather than only pull-out values. In addition, because we did not observe intraoperative complications such as screw pull-out and bending, we could not analyze the predicted forces in cases with complications. However, in this study, the forces generated at the bonescrew interface (peak 497 N) were lower than the thoracic pedicle screws pull-out forces of approximately 800 N reported in experimental studies (Liljenqvist et al., 2001). The present study validated only the geometrical aspects, and more investigations are needed to validate the model in terms of forces at the bonescrew interfaces. However, the implants tested by Liljenquist et al. were monoaxial pedicle screws which were different from the screws in the current study (poly-axial screws). Bonescrew forces were higher for monoaxial screws than polyaxial screws, indicating that in patients with large and stiff spinal deformities or in patients with compromised bone quality, screws with more degrees of freedom would offer better perspective to reduce bonescrew connection failure (Wang et al., 2012). Although the thresholds may serve as a comparison in the present study, therefore, those simulated cases exceeding the threshold may not be considered necessarily unsafe, it is likely that the anatomical correction technique may be used safely.

Our study has some limitations. First, we applied boundary conditions only to the pelvis and did not include the cervical spine, ribs, and scapulae. Although this simplification of the real spine represents a condition wherein the vertebral levels are not entirely fixed, including the cervical vertebrae could demonstrate a more naturalistic behavior of the uninstrumented spinal segment (Majdouline et al., 2012). Second, we only simulated one diameter although the screw diameter is known to have the highest effect on the force to failure compared to screw length (Cho et al., 2010; Bianco et al., 2019). Since this was a first phase study to simulate the surgical procedure, the simulation was maintained as simple as possible. Additionally, model validation was purely based on the final rod geometry and the main spinal curves. However, to ensure that the simulation model can predict postoperative alignment, a more detailed validation on single vertebra position and rotation, together with individualized screw’s models and trajectories, would be needed. Third, this study did not analyze other forces and moments such as medio-lateral forces and screw bending moments. The other loading components may play a role in other clinically relevant failure modes and may be addressed in the future. Finally, in this surgical simulation, preoperative curve flexibility was not considered, and distractioncompression force was not applied on each screw head. Nonetheless, the simulation model can predict postoperative surgical alignments. However, we are currently improving the model to incorporate both preoperative curve flexibility based on bending radiographs and distractioncompression procedures, as well as actual screw length and diameter. There may be a limit to improve the comparison between model prediction and actual postoperative correction; however, the incorporation may further improve the estimation of rod stress and screw force because this information will contribute to set stiffness matrix components and connections between the geometries.

## Conclusion

Our newly developed 4D planning simulation system incorporating pre-bent rods showed a significant correlation with the actual postoperative spinal alignment after anatomical 4D spinal correction surgery. The present study demonstrated the feasibility of our simulation system and the ability to simulate the surgical procedure using pre-bent rods. The FEA results in the simulation system measured for each individual patient would also provide a more realistic representation of the surgical procedures.

## Data Availability

The raw data supporting the conclusion of this article will be made available by the authors, without undue reservation.

## References

[B1] Abe Y. Ito M. Abumi K. Sudo H. Salmingo R. Tadano S. (2015). Scoliosis Corrective Force Estimation from the Implanted Rod Deformation Using 3D-FEM Analysis. Scoliosis 10, S2. 10.1186/1748-7161-10-S2-S2 25810754 PMC4331738

[B2] Abul-Kasim K. Ohlin A. (2014). Evaluation of Implant Loosening Following Segmental Pedicle Screw Fixation in Adolescent Idiopathic Scoliosis: a 2 Year Follow-Up with Low-Dose CT. Scoliosis 9, 13. eCollection 2014. 10.1186/1748-7161-9-13 25177357 PMC4149778

[B3] Argoubi M. Shirazi-Adl A. (1996). Poroelastic Creep Response Analysis of a Lumbar Motion Segment in Compression. J. Biomech. 29, 1331–1339. 10.1016/0021-9290(96)00035-8 8884478

[B4] Aubin C. E. Labelle H. Chevrefils C. Desroches G. Clin J. Eng A. B. M. (2008). Preoperative Planning Simulator for Spinal Deformity Surgeries. Spine 33, 2143–2152. 10.1097/BRS.0b013e31817bd89f 18794755

[B5] Belmont P. J. Jr Klemme W. R. Dhawan A. Polly D. W. Jr (2001). *In Vivo* Accuracy of Thoracic Pedicle Screws. Spine 26, 2340–2346. 10.1097/00007632-200111010-00010 11679819

[B6] Bianco R.-J. Arnoux P.-J. Mac-Thiong J.-M. Aubin C.-E. (2019). Thoracic Pedicle Screw Fixation under Axial and Perpendicular Loadings: A Comprehensive Numerical Analysis. Clin. Biomech. 68, 190–196. 10.1016/j.clinbiomech.2019.06.010 31238188

[B7] Cho W. Cho S. K. Wu C. (2010). The Biomechanics of Pedicle Screw-Based Instrumentation. The J. Bone Jt. Surg. Br. volume 92-B, 1061–1065. 10.1302/0301-620X.92B8.24237 20675747

[B8] Cidambi K. R. Glaser D. A. Bastrom T. P. Nunn T. N. Ono T. Newton P. O. (2012). Postoperative Changes in Spinal Rod Contour in Adolescent Idiopathic Scoliosis. Spine 37, 1566–1572. 10.1097/BRS.0b013e318252ccbe 22426445

[B9] Clement J.-L. Chau E. Kimkpe C. Vallade M.-J. (2008). Restoration of Thoracic Kyphosis by Posterior Instrumentation in Adolescent Idiopathic Scoliosis. Spine 33, 1579–1587. 10.1097/BRS.0b013e31817886be 18552674

[B10] Cobetto N. Aubin C.-E. Parent S. (2020). Anterior Vertebral Body Growth Modulation. Spine (Phila Pa 1976) 45, E1203–E1209. 10.1097/BRS.0000000000003533 32341305

[B11] Di Silvestre M. Lolli F. Bakaloudis G. Maredi E. Vommaro F. Pastorelli F. (2013). Apical Vertebral Derotation in the Posterior Treatment of Adolescent Idiopathic Scoliosis: Myth or Reality? Eur. Spine J. 22, 313–323. 10.1007/s00586-012-2372-2 22868455 PMC3555611

[B12] Galbusera F. Bassani T. La Barbera L. Ottardi C. Schlager B. Brayda-Bruno M. (2015). Planning the Surgical Correction of Spinal Deformities: Toward the Identification of the Biomechanical Principles by Means of Numerical Simulation. Front. Bioeng. Biotechnol. 3, 178. 10.3389/fbioe.2015.00178 26579518 PMC4630605

[B13] Galbusera F. Cina A. Panico M. Bassani T. (2021). The Importance of Curve Severity, Type and Instrumentation Strategy in the Surgical Correction of Adolescent Idiopathic Scoliosis: an In Silico Clinical Trial on 64 Cases. Sci. Rep. 11, 1799. 10.1038/s41598-021-81319-z 33469069 PMC7815774

[B14] Hasegawa K. Okamoto M. Hatsushikano S. Shimoda H. Ono M. Homma T. (2017). Standing Sagittal Alignment of the Whole Axial Skeleton with Reference to the Gravity Line in Humans. J. Anat. 230, 619–630. 10.1111/joa.12586 28127750 PMC5382592

[B15] Kokabu T. Kanai S. Abe Y. Iwasaki N. Sudo H. (2018). Identification of Optimized Rod Shapes to Guide Anatomical Spinal Reconstruction for Adolescent Thoracic Idiopathic Scoliosis. J. Orthop. Res. 36, 3219–3224. 10.1002/jor.24118 30062779

[B16] Kokabu T. Sudo H. Abe Y. Ito M. Ito Y. M. Iwasaki N. (2016). Effects of Multilevel Facetectomy and Screw Density on Postoperative Changes in Spinal Rod Contour in Thoracic Adolescent Idiopathic Scoliosis Surgery. PLoS One 11, e0161906. 10.1371/journal.pone.0161906 27564683 PMC5001696

[B17] La Barbera L. Larson A. N. Rawlinson J. Aubin C.-E. (2021). In Silico patient-specific Optimization of Correction Strategies for Thoracic Adolescent Idiopathic Scoliosis. Clin. Biomech. 81, 105200. 10.1016/j.clinbiomech.2020.105200 33317937

[B18] Le Navéaux F. Aubin C.-E. Parent S. O. Newton P. Labelle H. (2017). 3D Rod Shape Changes in Adolescent Idiopathic Scoliosis Instrumentation: How Much Does it Impact Correction? Eur. Spine J. 26, 1676–1683. 10.1007/s00586-017-4958-1 28180978

[B19] Le Navéaux F. Larson A. N. Labelle H. Wang X. Aubin C.-É. (2016). How Does Implant Distribution Affect 3D Correction and Bone-Screw Forces in Thoracic Adolescent Idiopathic Scoliosis Spinal Instrumentation? Clin. Biomech. 39, 25–31. 10.1016/j.clinbiomech.2016.09.002 27639485

[B20] Liljenqvist U. Hackenberg L. Link T. Halm H. (2001). Pullout Strength of Pedicle Screws versus Pedicle and Laminar hooks in the Thoracic Spine. Acta Orthop. Belg. 67, 157–163. 11383294

[B21] Little J. P. Izatt M. T. Labrom R. D. Askin G. N. Adam C. J. (2013). An FE Investigation Simulating Intra-operative Corrective Forces Applied to Correct Scoliosis Deformity. Scoliosis 8, 9. 10.1186/1748-7161-8-9 23680391 PMC3680303

[B22] Lopez Poncelas M. La Barbera L. Rawlinson J. J. Crandall D. Aubin C. E. (2021). Credibility Assessment of Patient-specific Biomechanical Models to Investigate Proximal Junctional Failure in Clinical Cases with Adult Spine Deformity Using ASME V&V40 Standard. Comput. Methods Biomech. Biomed. Eng. 24, 1–11. 10.1080/10255842.2021.1968380 34427119

[B23] Majdouline Y. Aubin C.-E. Sangole A. Labelle H. (2009). Computer Simulation for the Optimization of Instrumentation Strategies in Adolescent Idiopathic Scoliosis. Med. Biol. Eng. Comput. 47, 1143–1154. 10.1007/s11517-009-0509-1 19669822

[B24] Majdouline Y. Aubin C.-E. Wang X. Sangole A. Labelle H. (2012). Preoperative Assessment and Evaluation of Instrumentation Strategies for the Treatment of Adolescent Idiopathic Scoliosis: Computer Simulation and Optimization. Scoliosis 7, 21. 10.1186/1748-7161-7-21 23181833 PMC3546007

[B25] Matsumoto M. Watanabe K. Hosogane N. Kawakami N. Tsuji T. Uno K. (2013). Postoperative Distal Adding-On and Related Factors in Lenke Type 1A Curve. Spine 38, 737–744. 10.1186/1471-2474-15-36610.1097/brs.0b013e318279b666 23104198

[B26] Oda I. Abumi K. Cunningham B. W. Kaneda K. McAfee P. C. (2002). An *In Vitro* Human Cadaveric Study Investigating the Biomechanical Properties of the Thoracic Spine. Spine 27, E64–E70. 10.1097/00007632-200202010-00007 11805710

[B27] Oda K. Ohba T. Hiroshi Y. Fujita K. Tanaka N. Koyma K. (2021). Factors Affecting Pedicle Screw Insertional Torque in Spine Deformity Surgery. Spine (Phila Pa 1976) 46, E932–E938. 10.1097/BRS.0000000000004021 34384093

[B28] Pasha S. Flynn J. (2018). Data-driven Classification of the 3D Spinal Curve in Adolescent Idiopathic Scoliosis with an Applications in Surgical Outcome Prediction. Sci. Rep. 8, 16296. 10.1038/s41598-018-34261-6 30389972 PMC6214965

[B29] Peng Y. X. Zheng Z. Y. Wang, Md W. g. Liu L. Chen, Md F. Xu, Md H. t. (2020). Relationship between the Location of Ligamentum Flavum Hypertrophy and its Stress in Finite Element Analysis. Orthop. Surg. 12, 974–982. 10.1111/os.12675 32489000 PMC7307228

[B30] Robitaille M. Aubin C.-É. Labelle H. (2009). Effects of Alternative Instrumentation Strategies in Adolescent Idiopathic Scoliosis: a Biomechanical Analysis. J. Orthop. Res. 27, 104–113. 10.1002/jor.20654 18634064

[B31] Salmingo R. A. Tadano S. Abe Y. Ito M. (2014). Influence of Implant Rod Curvature on Sagittal Correction of Scoliosis Deformity. Spine J. 14, 1432–1439. 10.1016/j.spinee.2013.08.042 24275616

[B32] Senteler M. Weisse B. Rothenfluh D. A. Snedeker J. G. (2016). Intervertebral Reaction Force Prediction Using an Enhanced Assembly of OpenSim Models. Comput. Methods Biomech. Biomed. Eng. 19, 538–548. 10.1080/10255842.2015.1043906 26031341

[B33] Shao K. Wang H. Li B. Tian D. Jing J. Tan J. (2018). Morphology-based Realization of a Rapid Scoliosis Correction Simulation System. Comput. Biol. Med. 94, 85–98. 10.1016/j.compbiomed.2018.01.004 29408001

[B34] Shin J. K. Lim B.-Y. Goh T. S. Son S. M. Kim H.-S. Lee J. S. (2018). Effect of the Screw Type (S2-Alar-Iliac and Iliac), Screw Length, and Screw Head Angle on the Risk of Screw and Adjacent Bone Failures after a Spinopelvic Fixation Technique: A Finite Element Analysis. PLoS One 13, e0201801. 10.1371/journal.pone.0201801 30114271 PMC6095501

[B35] Sudo H. Abe Y. Kokabu T. Ito M. Abumi K. Ito Y. M. (2016). Correlation Analysis between Change in Thoracic Kyphosis and Multilevel Facetectomy and Screw Density in Main Thoracic Adolescent Idiopathic Scoliosis Surgery. Spine J. 16, 1049–1054. 10.1016/j.spinee.2016.04.014 27114351

[B36] Sudo H. Abe Y. Kokabu T. Kuroki K. Iwata A. Iwasaki N. (2018). Impact of Multilevel Facetectomy and Rod Curvature on Anatomical Spinal Reconstruction in Thoracic Adolescent Idiopathic Scoliosis. Spine (Phila Pa 1976) 43, E1135–E1142. 10.1097/BRS.0000000000002628 29528999

[B37] Sudo H. Ito M. Abe Y. Abumi K. Takahata M. Nagahama K. (2014). Surgical Treatment of Lenke 1 Thoracic Adolescent Idiopathic Scoliosis with Maintenance of Kyphosis Using the Simultaneous Double-Rod Rotation Technique. Spine 39, 1163–1169. 10.1097/BRS.0000000000000364 24732855

[B38] Sudo H. Ito M. Kaneda K. Shono Y. Abumi K. (2013). Long-Term Outcomes of Anterior Dual-Rod Instrumentation for Thoracolumbar and Lumbar Curves in Adolescent Idiopathic Scoliosis. J. Bone Jt. Surg. 95, e49. 10.2106/JBJS.L.0078110.2106/JBJS.L.00781 23595075

[B39] Sudo H. Tachi H. Kokabu T. Yamada K. Iwata A. Endo T. (2021). *In Vivo* deformation of Anatomically Pre-bent Rods in Thoracic Adolescent Idiopathic Scoliosis. Sci. Rep. 11, 12622. 10.1038/s41598-021-92187-y 34135445 PMC8209019

[B40] Ulrich D. van Rietbergen B. Weinans H. Rüegsegger P. (1998). Finite Element Analysis of Trabecular Bone Structure: a Comparison of Image-Based Meshing Techniques. J. Biomech. 31, 1187–1192. 10.1016/s0021-9290(98)00118-3 9882053

[B41] Wang X. Aubin C.-E. Crandall D. Parent S. Labelle H. (2012). Biomechanical Analysis of 4 Types of Pedicle Screws for Scoliotic Spine Instrumentation. Spine 37, E823–E835. 10.1097/BRS.0b013e31824b7154 22310096

[B42] Wang X. Boyer L. Le Navéaux F. Schwend R. M. Aubin C.-E. (2016). How Does Differential Rod Contouring Contribute to 3-dimensional Correction and Affect the Bone-Screw Forces in Adolescent Idiopathic Scoliosis Instrumentation? Clin. Biomech. 39, 115–121. 10.1016/j.clinbiomech.2016.10.002 27750078

[B43] Yamada K. Sudo H. Iwasaki N. Chiba A. (2020). Mechanical Analysis of Notch-free Pre-bent Rods for Spinal Deformity Surgery. Spine (Phila Pa 1976) 45, E312–E318. 10.1097/BRS.0000000000003269 31574057

[B44] Zhou Q.-k. Zeng F.-h. Tu J.-l. Dong Z.-q. Ding Z.-H. (2020). Influence of Cement-Augmented Pedicle Screw Instrumentation in an Osteoporotic Lumbosacral Spine over the Adjacent Segments: a 3D Finite Element Study. J. Orthop. Surg. Res. 15, 132. 10.1186/s13018-020-0165010.1186/s13018-020-01650-5 32264901 PMC7137326

