# Supplementary material for: Surgical Outcome Prediction Using a Four-Dimensional Planning Simulation System With Finite Element Analysis Incorporating Pre-bent Rods in Adolescent Idiopathic Scoliosis: Simulation for Spatiotemporal Anatomical Correction Technique
Source: Front Bioeng Biotechnol. 2021 Oct 12;9:746902. doi: 10.3389/fbioe.2021.746902 (PMC8546212; doi:10.3389/fbioe.2021.746902)
Supplement: Supplementary file 1 [file DataSheet1.PDF]

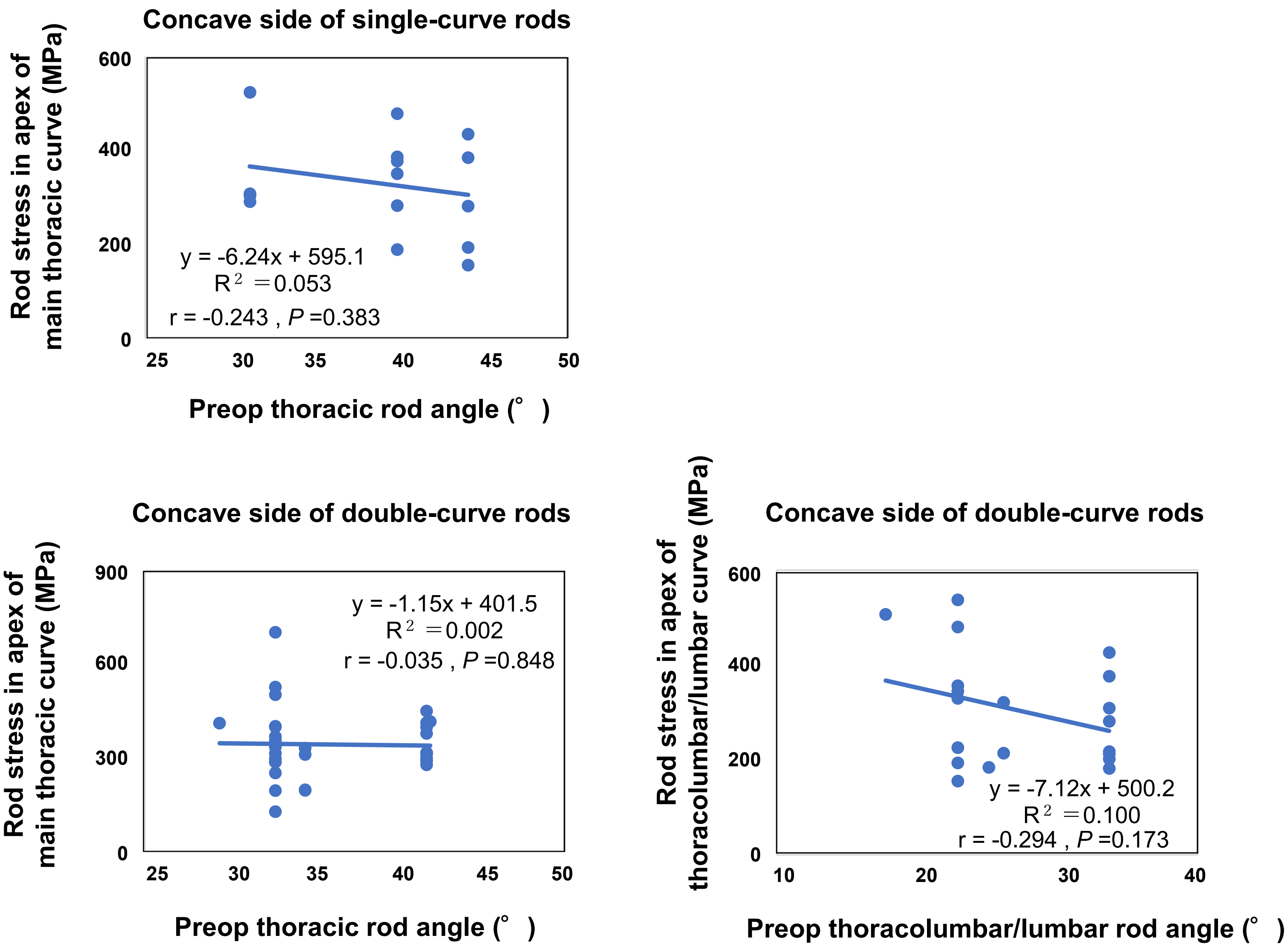

Supplementary figure 1. Correlation analysis between the preoperative rod angle and the rod stress in apex of main thoracic curve or thoracolumbar/lumbar curve.

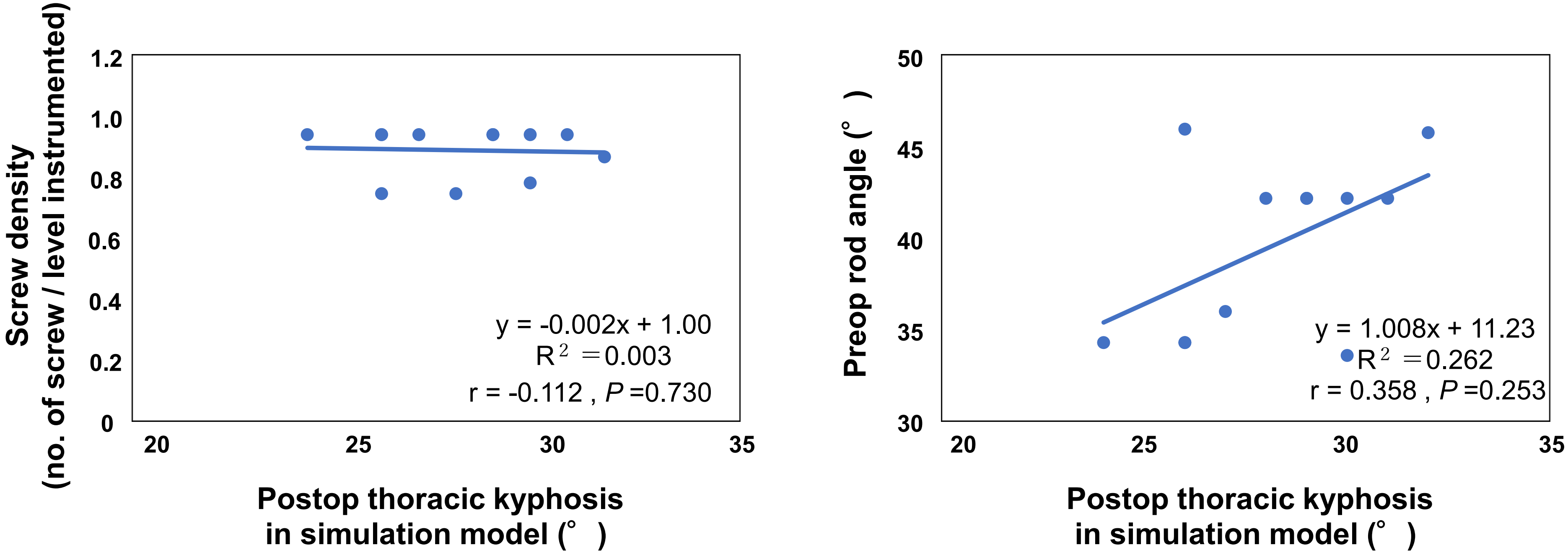

Supplementary figure 2. Correlation analysis between postoperative thoracic kyphosis in the simulation model and screw density on the concave side or preoperative rod angle in patients with hypothoracic (thoracic kyphosis < 15°) Lenke 1 curves (n=12).
